# Dignity and Distress towards the End of Life across Four Non-Cancer Populations

**DOI:** 10.1371/journal.pone.0147607

**Published:** 2016-01-25

**Authors:** Harvey Max Chochinov, Wendy Johnston, Susan E. McClement, Thomas F. Hack, Brenden Dufault, Murray Enns, Genevieve Thompson, Mike Harlos, Ronald W. Damant, Clare D. Ramsey, Sara Davison, James Zacharias, Doris Milke, David Strang, Heather J. Campbell-Enns, Maia S. Kredentser

**Affiliations:** 1 Department of Psychiatry, College of Medicine, Faculty of Health Sciences, University of Manitoba, Winnipeg, Canada; 2 Manitoba Palliative Care Research Unit, CancerCare Manitoba, Winnipeg, Canada; 3 Neurology, Department of Medicine, Faculty of Medicine, University of Alberta, Edmonton, Canada; 4 College of Nursing, Faculty of Health Sciences, University of Manitoba, Winnipeg, Canada; 5 George and Fay Yee Center for Healthcare Innovation, Department of Community Health Sciences, University of Manitoba, Winnipeg, Canada; 6 Winnipeg Regional Health Authority, Palliative Care Program, Winnipeg, Canada; 7 Division of Pulmonary Medicine, University of Alberta, Edmonton, Canada; 8 Department of Internal Medicine, College of Medicine, Faculty of Health Sciences, University of Manitoba, Winnipeg, Canada; 9 Community Health Sciences, University of Manitoba, Winnipeg, Canada; 10 Department of Medicine, University of Alberta, Edmonton, Alberta, Canada; 11 Section of Nephrology, College of Medicine, Faculty of Health Sciences, University of Manitoba, Winnipeg, Canada; 12 CapitalCare, Edmonton, Canada; 13 Faculty of Nursing, University of Alberta, Edmonton, Canada; 14 Department of Psychology, University of Alberta, Edmonton, Canada; 15 Geriatric Medicine, University of Manitoba, Winnipeg, Canada; 16 Geriatrics Program, Winnipeg Regional Health Authority, Winnipeg, Canada; 17 Interdisciplinary Cancer Control, Faculty of Health Sciences, Faculty of Graduate Studies, University of Manitoba, Winnipeg, Canada; 18 Department of Psychology, University of Manitoba, Winnipeg, Canada; University of Glasgow, UNITED KINGDOM

## Abstract

**Objective:**

The purpose of this study was to identify four non-cancer populations that might benefit from a palliative approach; and describe and compare the prevalence and patterns of dignity related distress across these diverse clinical populations.

**Design:**

A prospective, multi-site approach was used.

**Setting:**

Outpatient clinics, inpatient facilities or personal care homes, located in Winnipeg, Manitoba and Edmonton, Alberta, Canada.

**Participants:**

Patients with advanced Amyotrophic Lateral Sclerosis (ALS), Chronic Obstructive Pulmonary Disease (COPD), End Stage Renal Disease (ESRD); and the institutionalized alert frail elderly.

**Main Outcome Measure:**

In addition to standardized measures of physical, psychological and spiritual aspects of patient experience, the Patient Dignity Inventory (PDI).

**Results:**

Between February 2009 and December 2012, 404 participants were recruited (ALS, 101; COPD, 100; ESRD, 101; and frail elderly, 102). Depending on group designation, 35% to 58% died within one year of taking part in the study. While moderate to severe loss of sense of dignity did not differ significantly across the four study populations (4–11%), the number of PDI items reported as problematic was significantly different i.e. ALS 6.2 (5.2), COPD 5.6 (5.9), frail elderly 3.0 (4.4) and ESRD 2.3 (3.9) [p < .0001]. Each of the study populations also revealed unique and distinct patterns of physical, psychological and existential distress.

**Conclusion:**

People with ALS, COPD, ESRD and the frail elderly face unique challenges as they move towards the end of life. Knowing the intricacies of distress and how they differ across these groups broadens our understanding of end-of-life experience within non-cancer populations and how best to meet their palliative care needs.

## Background

Despite their physical, psychological and existential suffering, patients with non-malignant conditions such as ALS, ESRD, and COPD tend to be underserved by palliative care[1–7]. Such is also the case for the institutionalized elderly, for whom palliative care services and supports are frequently lacking[6].The elderly do not usually think of themselves as terminally ill [8]; while patients with life limiting illnesses often manage to disregard the reality of their terminal circumstances[9–11]. Relative to cancer, people with non-malignant conditions tend to have less prognostic certainty. While the combination of uncertainty and denial may curtail the application of palliative approaches, so too does lack of familiarity with the burden of suffering people face as they move closer to death.

The lived experience of end stage cancer has been well documented, [12–14] as have the physical, psychological, existential and spiritual sources of distress that can undermine dignity in patients with advanced cancer. The latter derives from a study of various factors influencing sense of dignity in dying patients; culminating in an empirical model of dignity in the terminally ill [15]. This model is unique, in that it provides therapeutic guidance and insight into how dignity can be maintained or undermined as patients draw closer to death. This model forms the basis of dignity conserving palliative care [16], given that each of the model themes and sub-themes implicate issues that clinicians must be aware of and attentive to, in order to mitigate patient suffering and distress. The model also informed the development a psychometric coined the Patient Dignity Inventory (PDI)[17]. The PDI is a novel screening instrument tapping into multiple sources of distress salient for patients with limited life expectancies[17]. The PDI has been validated in multiple languages and applied in various palliative care settings and studies worldwide [18–24]. One national study reported that amongst patients with varying stages of cancer, the PDI readily helped clinicians identify dignity related distress. In 76% of instances, it disclosed information clinicians were previously unaware of and enabled them to provide more timely and targeted therapeutic responses to patients’ concerns[25]. To date, however, this research has largely been confined to cancer. The objective of this study was to identify four non-cancer populations that might benefit from a palliative approach and, using the PDI, describe and compare the landscape of dignity related distress for each of these diverse groups.

## Methods

A prospective multi-site approach was used to examine physical, psychological, existential and spiritual issues facing patients with advanced ALS, COPD, ESRD; and the institutionalized alert frail elderly (subsequently referred to as the frail elderly). This report describes cross-sectional patient/resident data and differences across these diverse groups. Participating centres included Winnipeg, Canada (Health Sciences Centre, St. Boniface General Hospital, Winnipeg Regional Health Authority) and Edmonton, Canada (University of Alberta Hospital, Alberta Health Services, Covenant Health, and CapitalCare). The study protocol was approved by ethics boards at the University of Manitoba and the University of Alberta.

Clinical staff in outpatient clinics, inpatient facilities or personal care homes identified potential participants according to study entry criteria. Study participants were within one of the four clinical populations of interest, able to read and understand English, and competent (based on clinical consensus). Eligibility criteria within each of the four study populations were designed to identify people whose current clinical status suggested imminently life-limiting circumstances, hence most likely to benefit from a palliative care approach. These criteria were also designed to distinguish groups from one another; i.e. participants were only able to meet eligibility criteria for one group. Patients with ALS were considered eligible if they had: 1) a medically confirmed diagnosis of their condition; 2) mobility limitations, dysphagia, dyspnea or speech problems that interfered with their social or occupational functioning; and 3) were between 18 and 80 years of age. Patients with ESRD were eligible if they were: 1) receiving dialysis for 3 or more months; and 2) between 65 and 80 years of age. Eligibility criteria for patients with COPD included: 1) having stage 4 disease, based on Global Initiative for Chronic Obstructive Lung Disease GOLD classification[26]; and 2) being between 65 and 80 years of age. The frail elderly were defined as: 1) being over 80 years of age; 2) residing in a personal care home (PCH); and 3) requiring assistance with two or more basic activities of daily living (bathing, dressing, toileting, grooming, feeding, ambulation); and 4) having a Cognitive Performance Scale (CPS) of zero to three (i.e. none to mild cognitive decline) [27].

All patients or residents meeting eligibility criteria were asked permission by the clinical staff to have their name released to the study nurse or research assistant, who then confirmed eligibility and obtained written consent. Basic demographic information was gathered, along with information regarding their current health status. Participants were then administered questionnaires by the research nurse or assistant–either in the hospital, outpatient unit or personal care home—assessing their existential distress (Structured Interview of Symptoms and Concerns [SISC][28]); hope (Herth Hope Index [HHI] [29]); spirituality (Spiritual Survey [30]); physical symptoms (Revised Edmonton Symptom Assessment Scale [ESAS-R] [31]); comorbidities (Charlson Comorbidity Index [32]); activities of daily living (The Katz Index of Daily Basic Living [33]); depression (using the depression subscale of the Hospital Anxiety Depression Scale [HADS] [34]); and social support (The Multidimensional Scale of Perceived Social Support [MSPSS] [35]).

Participants were also administered the Patient Dignity Inventory (PDI), a 25-item instrument tapping into a broad spectrum of physical, psychological and existential issues, with each item ranging from 1 (not a problem) to 5 (an overwhelming problem) [a score of ≥3 denotes *a problem*]. The protocol was very well tolerated across all study groups; (details regarding the latter, including attitudes towards being research participants, will be reported in a separate manuscript). In preparing to launch the study and to elicit feedback regarding the applicability of this instrument to non-cancer populations, the PDI was administered to 62 participants (13 with ALS; 21 with ESRD; 16 with COPD; and 12 frail elderly). On the basis of this pilot data, two items that made reference to *sickness* were revised to read *health status*, given that the frail elderly did not necessarily see themselves as sick.

### Analysis

One hundred patients were recruited for each of four population groups. Using estimation procedures established by Cohen [36], this sample size ensures an 80 per cent power of detecting a small to medium-sized difference between the four groups when testing is carried out at the conventional 0.05 level. All analyses were conducted using SAS Version 9.3 (SAS Institute, Cary NC). Descriptive statistics (mean, standard deviation, and percentages, as appropriate) were calculated. The proportion of individuals experiencing a particular problem, defined as a PDI item rating of ≥ 3, was compared between groups using the Chi-squared test or the Fisher’s exact test. The total number of PDI problems was compared between groups using ANOVA models (PROC MIXED in SAS) generalized to allow heterogeneous group variances. The Kruskal-Wallis test was used to compare the distributions of ESAS items, Spirituality Survey, HADS, MSPSS, and the HHI between patient groups, as well as between those reporting intact (SISC Dignity Rating < 3) versus fractured sense of dignity (SISC Dignity Rating ≥ 3). Unless specifically stated, all statistical tests were carried out on a two-tailed basis, using 0.05 alpha level of significance.

## Results

Study recruitment took place between February 2009 and December 2012. Date of death was tracked until September 2013. Of 663 eligible people approached, 249 declined. Reasons for non-participation included not interested (222), too busy (14), did not respond to an invitation to take part (11), or the family said no (2). Of the remaining 404 participants (61%), 101 had ALS, 100 COPD, 101 ESRD and 102 were frail elderly (Table 1). While there was some variation within groups, the total sample was evenly divided between the two primary recruitment sites (see Table 1). Fifty-two percent of the sample was male. Average ages were 63.9 (12.0) years for ALS, 72.3 (4.8) years for COPD, 72.3 (4.4) years for ERSD and 88.2 (5.0) years for the frail elderly. By September 2013, 45% of participants had died (ALS, 58% [1.13 (.87) year survival]; frail elderly, 42% [1.49 (.95)]; ESRD, 41% [1.6 (.93)]; and COPD, 35% [1.10 (.87)]).

**Table 1:** Demographics of Study Population [N(%)].

Patient Group		ALS	COPD	ESRD	FE	N	Total (%)
		(N = 101)	(N = 100)	(N = 101)	(N = 102)	404	100
**Age (mean years)**		63.9 (12.0)	72.3 (4.8)	72.3 (4.4)	88.2 (5.0)		
**City**	Edmonton	63 (62.4)	49 (49.0)	51 (50.5)	42 (41.2)	**205**	**50.7**
	Winnipeg	38 (37.6)	51 (51.0)	50 (49.5)	60 (58.8)	**199**	**49.3**
**Marital Status**	Married or common-law	69 (68.3)	51 (51.0)	51 (50.5)	14 (13.7)	**185**	**45.8**
	Widow/er	9 (8.9	21 (21)	28 (27.7)	73 (71.6)		
	Other	23 (22.8)	28 (28.0)	22 (21.8)	15 (14.7)	**219**	**54.2**
**Gender**	Male	68 (64.8)	40 (40.4)	60 (58.8)	46 (43.4)	**210**	**52.08**
	Female	37 (35.2)	59 (59.6)	42 (41.2)	60 (56.6)	**194**	**48**
**Current Residence**	At home	88 (87.1)	84 (84.9)	91 (90.1)	0 (0.0)	**263**	**65.3**
	Personal Care or Nursing Home	1 (1.0)	1 (1.0)	7 (6.9)	78 (76.5)	**87**	**21.6**
	Long term care facility	6 (5.9)	9 (9.1)	1 (1.0)	24 (23.5)	**40**	**9.9**
	Hospice	4 (4.0)	0 (0.0)	0 (0.0)	0 (0.0)	**4**	**1**
	Other	2 (2.0)	5 (5.1)	2 (2.0)	0 (0.0)	**9**	**2.2**
**Living With**	Spouse/Partner	68 (65.4)	50 (51.0)	50 (49.5)	4 (3.9)	**172**	**42.6**
	Other Residents	11 (10.9)	10 (10.0)	7 (6.9)	95 (93.1)	**123**	**30.5**
	Alone	15 (14.9)	30 (30.0)	28 (27.7)	4 (3.9)	**77**	**19.1**
	Children	14 (13.9)	12 (12.0)	19 (18.8)	0 (0.0)	**45**	**11.1**
	Other Relative	3 (2.9)	2 (2.0)	5 (5.0)	0 (0.0)	**10**	**2.5**
	Sibling	3 (3.0)	1 (1.0)	1 (1.0)	0 (0.0)	**5**	**1.2**
	Parents	2 (2.0)	0 (0.0)	0 (0.0)	0 (0.0)	**2**	**0.5**
	Friend	0 (0.0)	0 (0.0)	2 (2.0)	0 (0.0)	**2**	**0.5**
	Other	4 (4.0)	3 (3.0)	1 (1.0)	0 (0.0)	**8**	**2**
**Primary Social**	Children	34 (34.0)	52 (52.0)	55 (54.5)	68 (66.8)	**209**	**51.9**
**Support**	Spouse/Partner	67 (67.0)	52 (52.0)	48 (47.5)	10 (9.8)	**177**	**43.9**
	Friend	20 (20.4)	23 (23.0)	31 (30.7)	13 (12.9)	**87**	**21.8**
	Other Relative	8 (8.0)	11 (11.0)	15 (14.9)	20 (19.6)	**54**	**13.4**
	Sibling	16 (16.0)	11 (11.0)	9 (8.9)	13 (12.8)	**49**	**12.2**
	No one	0 (0.0)	3 (3.0)	4 (4.0)	1 (1.0)	**8**	**2**
	Parent	4 (4.0)	1 (1.0)	0 (0.0)	0 (0.0)	**5**	**1.2**
	Other	11 (11.0)	4 (4.0)	5 (5.0	11 (10.8)	**31**	**7.7**
**Religion**	Protestant	19 (19.0)	24 (24.0)	37 (37.0)	29 (28.4)	**109**	**27.1**
	Roman Catholic	29 (29.0)	29 (29.0)	25 (25.0)	22 (21.6)	**105**	**26.1**
	None	24 (24.0)	25 (25.5)	15 (15.0)	18 (17.7)	**82**	**20.4**
	Jewish	0 (0.0)	0 (0.0)	2 (2.0)	9 (8.8)	**11**	**2.7**
	Muslim	1 (1.0)	0 (0.0)	2 (2.0)	0 (0.0)	**3**	**0.8**
	Other	27 (27.0)	22 (22.0)	19 (19.0)	24 (23.5)	**92**	**22.9**
**Education Completed**	Less than High School	23 (23.0)	46 (46.0)	45 (44.6)	47 (46.1)	**161**	**39.9**
	High School Complete	23 (23.0)	19 (19.0)	19 (18.8)	13 (12.8)	**74**	**18.4**
	Greater than High School	54 (54.0)	36 (36.0)	37 (36.6)	42 (41.2)	**168**	**41.6**
**Annual Income (net last 12 months)**	≤ 60k	50 (49.5)	71 (71.0)	73 (72.27)	50 (49.0)	**244**	**60.4**
	≥ 60k	24 (23.8)	8 (8.0)	16 (15.84)	6 (5.9)	**54**	**13.4**
	No answer	27 (26.7)	21 (21.0)	12 (11.88)	46 (45.1)	**106**	**26.2**

### Social Support

Between 84–90% of study participants in the ALS, COPD and ESRD cohorts were living at home, while all of the frail elderly subjects were living in residential care. Within the ALS, COPD and ESRD cohorts, 51.0–68.3% were married or common-law; however, only 13.7% of the frail elderly were married or common-law (the majority [71.6%] were widowed). Similarly, while primary social support from spouse/partner in other groups ranged between 47.5–67.0%, only 9.8% of the frail elderly identified spouse/partner as their primary support, with children fulfilling that role in 66.8% of instances. The perceived level of social support, as measured by the MSPSS, ranged from least supported, the frail elderly, followed by ESRD, COPD, to the most supported, ALS (p < .0001) (Table 2). Relative to other groups, the frail elderly felt less supported by family (p = .005), friends (p < .0001) or a significant other (p < .0009).

**Table 2:** Scores on Measures of Functioning Across the Four Study Groups; and Mean Levels of Functioning by Level of Dignity. Superscript indicates ranking of functioning between groups [1 = highest, to 4 = lowest]

		Level of Distress Across Study Groups [Mean (SD)]					SISC DIGNITY	SISC DIGNITY	
							<3 (Intact)	> 3 (Fractured)	
		ALS	COPD	ESRD	FE	p-value^+^	Means (SD)	Means (SD)	p-value^+^
**Mean Number of PDI Items Rated Problematic**		6.2 (5.2)^4^	5.6 (5.9)^3^	2.3 (3.9)^1^	3.0 (4.4)^2^	< .0001	3.9 (4.9)	9.2 (5.6)	< .0001
**ESAS-R Items**	Pain^a^	2.3 (2.6)^4^	3.5 (3.6)^1^	3.0 (3.2)^2^	2.7 (3.1)^3^	NS	2.8 (3.1)	4.1 (3.6)	-0.07
	Nausea^a^	0.5 (1.4)^4^	0.8 (1.8)^2^	1.0 (2.1)^1^	0.7 (1.6)^3^	NS	.07 (1.7)	1.0 (2.2)	NS
	Drowsiness^a^	2.1 (2.7)^3^	2.4 (2.9)^2^	2.5 (2.8)^1^	1.6 (2.4)^4^	NS	2.1 (2.7)	3.2 (2.9)	0.05
	Shortness of Breath^a^	2.9 (3.0)^2^	6.0 (3.0)^1^	1.5 (2.3)^3^	1.1 (1.9)^4^	< .0001	2.8 (3.2)	3.5 (3.8)	NS
	Anxiety^a^	2.4 (2.6)^2^	3.4 (3.4)^1^	1.7 (2.5)^3^	1.4 (2.3)^4^	< .0001	2.1 (2.7)	4.1 (3.6)	0.003
	Fatigue^a^	4.3 (2.9)^1^	4.2 (3.1)^2^	3.7 (3.1)^3^	2.6 (2.8)^4^	< .0001	3.6 (3.0)	6.0 (3.3)	0.001
	Constipation^a^	1.8 (2.8)^1^	1.6 (2.9)^2^	1.4 (2.6)^3^	1.8 (2.8)^1^	NS	1.5 (2.6)	3.8 (3.7)	0.0002
	Diarrhea^a^	0.5 (1.5)^3^	0.5 (1.8)^3^	0.9 (1.9)^1^	0.7 (2.0)^2^	0.02	.65 (1.8)	.80 (2.3)	NS
	Weakness^a^	5.6 (3.3)^1^	3.5 (3.1)^2^	3.2 (3.0)^3^	2.6 (2.9)^4^	< .0001	3.5 (3.2)	5.8 (3.4)	0.001
	Trouble sleeping^a^	1.9 (2.6)^3^	2.4 (3.4)^2^	3.2 (3.3)^1^	1.4 (2.5)^4^	0.0001	2.2 (3.0)	3.1 (3.6)	NS
	Dizziness^a^	0.6 (1.3)^3^	1.5 (2.5)^1^	0.9 (1.9)^2^	0.9 (2.0)^2^	0.04	.9 (2.0)	1.2 (1.7)	NS
	Difficulty thinking^a^	0.7 (1.5)^4^	0.9 (2.1)^3^	1.0 (2.0)^2^	1.3 (2.5)^1^	NS	.9 (2.0)	1.6 (2.4)	-0.07
	Will to live^b^	8.4 (2.9)^3^	9.2 (2.0)^1^	8.9 (2.3)^2^	8.4 (2.6)^3^	NS	8.7 (2.4)	8.0 (3.4)	NS
	Appetite^b^	6.5 (3.4)^4^	7.5 (3.0)^2^	8.0 (2.5)^1^	7.3 (2.8)^3^	0.009	7.4 (2.9)	5.7 (3.3)	0.006
	Activity^b^	4.0 (2.8)^4^	4.5 (2.7)^3^	6.0 (2.6)^1^	4.6 (2.9)^2^	< .0001	4.9 (2.8)	3.3 (3.5)	0.01
	Wellbeing^b^	7.3 (3.0)^4^	7.4 (2.7)^3^	7.8 (2.4)^2^	7.9 (2.6)^1^	NS	7.8 (2.5)	4.9 (3.2)	< .0001
**No. ADL Dependencies**^e^		2.2 (2.1)^2^	0.7 (1.2)^3^	0.4 (1.0)^4^	3.3 (1.5)^1^	< .0001	1.6 (1.8)	2.9 (2.2)	0.002
**Charlson Comorbidity**^f^		2.5 (1.5)^4^	5.3 (1.9)^3^	7.8 (2.0)^1^	7.1 (1.6)^2^	< .0001	4.6 (2.5)	5.8 (2.7)	0.045
**Hope Hearth Index (HHI)**^c^		37.4 (4.7)^3^	37.8 (5.0)^2^	39.0 (5.5)^1^	35.9 (6.0)^4^	0.008	37.8 (5.2)	34.0 (6.1)	0.0008
**Spirituality Belief Scale**^d^		44.7 (19.5)^3^	43.9 (20.1)^4^	49.3 (22.2)^1^	48.4 (19.8)^2^	NS	47.2 (20.3)	37.8 (22.0)	0.02
**HADS**^g^		12.6 (3.6)^1^	12.5 (3.7)^2^	10.8 (3.1)^4^	11.6 (3.4)^3^	0.0004	11.7 (3.4)	14.5 (4.6)	0.002
**MSPSS Social Support**^h^		74.6^1^ (10.5)^1^	69.6 (12.0)^2^	69.4 (11.8)^3^	66.8 (12.4)^4^	< .0001	70.2 (11.9)	69.3 (14.0)	0.01

^+^Kruskal-Wallis Comparison of Distributions

^a^ESAS-R item; scored 0 (no problem) to 10 (very significant problem)

^b^ESAS-R item; scored 0 (low/poor) to 10 (high/good)

^c^Hope Hearth Index; Scored 12 (least hope) to 48 (most hope)

^d^Spiritual Beliefs Scale; Scored 9 (unimportant/not worthwhile/rarely) to 90 (very important/very worthwhile/always)

^e^The Katz Index of Daily Basic Living; Scored 0 (independent in six functions) to 6 (dependent in six functions)

^f^Charlson Comorbidity Index; Number of Comorbid conditions

^g^HADS (depression items only); Scored 0 (normal/no depression) to 21 (severe depression)

^h^MSPSS (Multidimensional Scale of Perceived Social Support); Scored 12 (low social support) to 84 (high social support). 1, 2, 3, 4 Indicates ranking of prevalence of PDI problem, from most prevalent (1) to least prevalence (4)

### Physical Disability and Symptom Burden

There were no significant differences across groups on measures of pain, nausea, drowsiness, constipation and difficulty thinking (Table 2). Fatigue and weakness were most pronounced in ALS (p<0.001), as were lack of appetite (p < .009) and activity (p < .0001). Fatigue in COPD appeared comparable to ALS, with COPD patients most likely to experience shortness of breath (p < .001) and dizziness (p < .04). Patients with ESRD reported the most trouble sleeping (p < .0001), diarrhea (p < .02) and the highest number of co-morbidities (7.8, [2.0]; p < .0001). The frail elderly reported the most dependencies (3.3, [1.5]; p < .0001).

### Existential, Spiritual and Psychological Distress

Mean ESAS-R ratings on will to live and sense of well-being were comparable between groups, as were measures of spiritual beliefs (Table 2). Based on SISC measures (Table 3), the four groups did not differ with respect to sense of suffering (5.9–12.8%), hopelessness (4.0–8.7%) or general dissatisfaction with life (2.0–5.2%). The only group to report moderate to extreme suicidal ideation was ESRD (5.9%: p = .0005).(Table 3). The highest rate of anxiety was reported in patients with COPD, as measured by the ESAS-R (Table 2) and the PDI (Table 4). Depression as measured by the HADS (Table 2) and the PDI (Table 4), appeared to be significantly higher in COPD and ALS relative to the other groups. The frail elderly reported the highest prevalence of moderate to extreme desire for death (7.9%; p = .04) (Table 3); and the lowest level of hope (based on the HHI; Table 2 [p = .008]).

**Table 3:** Percentage of Participants with Existential Distress Across Study Groups; and by SISC Dignity Ratings.

SISC Item	Distress Across Study Groups					SISC DIGNITY	SISC DIGNITY	p-value^+^
						<3 (Intact)	>3 (Fractured)	
	*(Percentage of participants with moderate to extreme [SISC item score* *≥3] distress)*							
	ALS	COPD	ESRD	FE	p-value	Percentage	Percentage	
	(N = 100)	(N = 100)	(N = 101)	(N = 100)				
Loss of Dignity	11.01	4.03	4.03	6.02	NS	NA	NA	N/A
Suffering	12.81	10.23	5.94	12.02	NS	8.3	36	< .0001
Hopelessness	8.71	8.22	4.04	5.03	NS	5.1	29.2	< .0001
Desire for death	2.92	2.03	1.04	7.91	0.04	2.4	16	0.0002
Suicidal	0	0	5.91	0	0.0005	1.4	4	NS
General Dissatisfaction	3.03	5.21	4.02	2.04	NS	2.2	24	< .0001

^+^Pearson Chi-square. SISC items scored on 0 (none) to 6 (extreme)

**Table 4:** Percentage of Participants with Individual PDI Problems (>3) Across Four Study Groups; and by SISC Dignity Ratings.

Patient Dignity Inventory (PDI)	ALS	COPD	ESRD	FE	p-value^+^	SISC DIGNITY	SISC DIGNITY	p-value^+^
	(N = 101)	(N = 100)	(N = 101)	(N = 101)		< 3 (Intact)	>3 (Fractured)	
						N = 375	N = 25	
Not being able to carry out tasks associated with daily living	**45.51**	33.03	9.94	**34.92**	< .0001	28.7	52	0.01
Not being able to attend to my bodily functions independently	26.72	10.03	2.04	**31.71**	< .0001	15.2	44	0.0002
Experiencing physically distressing symptoms	**42.62**	**60.61**	**24.84**	**26.53**	< .0001	37.8	44	NS
Feeling that how I look to others has changed significantly.	22.81	20.02	2.04	5.93	< .0001	11.2	32	0.002
Feeling depressed.	22.02	23.01	7.94	10.83	< .001	14.4	36	0.004
Feeling anxious.	24.82	30.01	10.04	12.83	< .0001	17.9	40.4	0.007
Feeling uncertain about my health and health care.	36.62	**44.41**	14.93	12.84	< .0001	25.6	48	0.01
Worrying about my future.	41.61	29.02	9.94	10.93	< .0001	20.5	56	< .0001
Not being able to think clearly.	5.92	9.01	5.92	5.03	NS	6.4	8	NS
Not being able to continue with my usual routines.	**49.51**	**49.02**	**18.83**	18.64	< .0001	32.2	60	0.005
Feeling like I am no longer who I was.	41.51	37.42	14.93	13.94	< .0001	24.6	60	0.0001
Not feeling worthwhile or valued.	24.71	24.22	9.04	10.83	< .001	16	32	0.04
Not being able to carry out important roles	42.01	28.02	12.93	12.84	< .0001	22.6	44	0.02
Feeling that life no longer has meaning or purpose.	15.82	19.21	7.94	12.93	NS	12.6	36	0.0011
Feeling that I have not made a meaningful and/or lasting contribution in my life	7.01	6.02	5.03	2.94	NS	4.8	12	NS
Feeling that I have ‘unfinished business’	32.71	17.02	13.13	5.94	< .0001	15	48	< .0001
Concern that my spiritual life is not meaningful.	2.03	4.02	4.02	4.91	NS	3.7	4	NS
Feeling that I am a burden to others.	**42.61**	23.22	10.94	12.93	< .0001	19.5	60	< .0001
Feeling that I don’t have control over my life.	37.61	28.32	**16.83**	16.74	< .001	22.1	64	<. 0001
Feeling that my health and care needs have reduced my privacy.	26.71	16.02	10.04	14.93	< .01	14.7	48	< .0001
Not feeling supported by my community of friends and family.	2.02	8.01	2.02	2.02	< .05	2.9	12.5	0.01
Not feeling supported by my health care providers.	2.03	5.02	6.91	5.02	NS	4	16.7	0.005
Feeling like I am no longer able to mentally cope with challenges to my health.	7.92	10.21	4.04	7.13	NS	6.4	21	0.009
Not being able to accept the way things are.	12.02	16.21	6.04	10.03	NS	9.4	34.8	0.0001
Not being treated with respect or understanding by others.	5.92	10.01	4.04	5.03	NS	5.9	12	NS

^**+**^Pearson Chi-square. The three most prevalent PDI items within each group appear in bold.

### Dignity related distress

While there were no significant differences across the four groups on global ratings of moderate to extreme loss of dignity (SISC item score > 3; Table 3)—ALS, 11.0%; frail elderly, 6.0%; and COPD and ESRD, both 4.0%—participants varied significantly on the mean number of PDI items rated as problematic: ALS 6.2 (5.2), COPD 5.6 (5.9), frail elderly 3.0 (4.4) and ESRD 2.3 (3.9) [p < .0001] (Table 2). Patients with ALS were significantly more likely than other groups to identify the following PDI items as problematic: not being able to continue my usual routines (49.5%); not being able to carry out tasks associated with daily living (45.5%); feeling a burden to others (42.6%); worrying about my future (41.6%); not being able to carry out important roles (42.0%); feeling like I am no longer who I was (41.5%); feeling that I don’t have control over my life (37.6%); feelings of unfinished business (32.7%); feeling my health can care needs have reduced my privacy (26.7%); not feeling worthwhile or valued (24.7%); and feeling how I look to others has changed significantly (22.8%) [p < .01-.0001]. Relative to the other groups, patients with COPD were most likely to rate the following PDI items as problematic: experiencing physically distressing symptoms (60.6%), uncertain about my health and health care (44.4%), feeling anxious (30.0%) or depressed (23.0% in COPD) (p < .001-.0001). Relative to other groups, frail elderly patients were most likely to identify ‘not being able to attend to my bodily functions independently’ as problematic (31.7%) [p < .0001]. ESRD did not rate significantly highest on any single PDI item (Table 4).

There were differences between participants whose dignity was intact and those whose dignity was fractured (Tables 2–4). Those whose dignity was intact reported fewer PDI items as problematic (3.9 [4.9]) relative to those whose dignity was fractured (9.2 [5.6], p < .0001). While study group affiliation, gender, marital status and age did not differentiate between these two groups, PDI items that most significantly differentiated these groups (p < .0001) included worrying about the future, feeling like I am no longer who I was, feelings of unfinished business, feeling a burden to others, not having control over my life, feeling that my health and care needs have reduced my privacy and not being able to accept the way things are (Table 4). Fractured dignity was also highly affiliated with intensity of suffering (p < .0001), hopelessness (p < .0001) desire for death (p = .0002) and decreased general life satisfaction and fulfillment (p < .0001)[Table 3]. Besides these dominantly existential issues, psychological symptoms such as anxiety (based on the PDI [p = .007] and the ESAS-R [p = .003]) and depression (based on the HADS [p = .002] and PDI [p = .004]) differentiated those with intact dignity from those whose dignity was fractured; various physical parameters, to a lesser extent, differentiated these two groups. Those reporting fractured dignity reported more dependencies (p = .002), fatigue (p = .001), constipation (p = .0002); weakness (p = .001) and poorer appetite (p = .0006) [Table 2].

## Discussion

This study is the first to describe and compare the landscape of dignity related distress across several non-cancer populations including advanced COPD, ALS, ESRD, and the frail institutionalized elderly. While overall loss of dignity did not differ significantly across these study populations and were comparable to those previously reported in end-stage cancer [37], the patterns of distress in each of these groups revealed distinct and important insights. Patients with ALS reported more dignity related distress such as not being able to fulfill important roles, tasks or daily routines; feeling like a burden to others, feeling a loss of control and no longer feeling worthwhile or valued. They experienced more weakness and fatigue, and were more likely to feel depressed, and to worry about the future. Their ratings of shortness of breath were second only to COPD, which is noteworthy, given the association in ALS between fear of suffocation and requests for euthanasia or physician-assisted death [38].

COPD patients rated on average 5.7 PDI items as problematic, identical to the number reported by hospice patients with advanced cancer [37].These patients were most likely to experience physically distressing symptoms and predictably, the highest intensity of shortness of breath. Consistent with clinical experience and the published literature [39], their intensity and frequency of anxiety were highest. The etiology of shortness of breath in COPD is thought be physiological and affective, with affected neurophysiological mechanisms causing distressing sensations, contributing to panic and anxiety [40]; and anxiety and depression intensifying sensations of shortness of breath [41].Low desire for death and the absence of suicidal ideation suggests that a slow insidious progression of illness allows for gradual adaptation to physical symptoms, disability and mounting losses. It also appears that limited prognostic awareness or prognostic disavowal form part of the psychological landscape, even for patients with advanced COPD [42, 43].Over the course of the study, patients with COPD displayed the lowest mortality (35%), but were most likely to express uncertainty about their health and health care. This is consistent with prior studies and prognostic models [44], underscoring the observation that patients with COPD have a less predictable terminal course, posing a barrier to the earlier initiation of palliative care [45].

While patients with ESRD did not report any PDI items in excess of the other study populations, they were the only group that included patients with moderate to severe suicidal ideation. These patients also reported the highest number of comorbidities and prominent symptom burden. Paucity of depression or loss of hope suggests that suicidal ideation may be driven by physical, more so than psychological factors. This is consistent with previous studies, which indicate that patients contemplating dialysis discontinuation do not seem to be influenced by major depression [46].The endorsement of suicidal ideation for this group may be a proxy for contemplating dialysis discontinuation [47].Decisions to withdraw dialysis now precede one in four deaths of patients who have end-stage renal disease [48].

The frail elderly are unique from the other study groups in several ways. Rather than being defined on the basis of illness, they were defined on the basis of age and frailty. While the elderly are not typically described as ‘terminally ill’, 42% of them died over the course of the study. Relative to the other groups, they are the most isolated and report the lowest social support from family, friends or a significant other. They report feeling the least hope, in the absence of significant worry about the future. The measure of hope (HHI) was based on a definition developed by Dufault and Martocchio [49], who described hope as a multidimensional construct that is characterized by confident yet uncertain expectations of achieving good, which is realistically possible and personally significant. This suggests that the elderly do not see their future holding such potential. Along with reporting the highest desire for death, in the absence of suicidal ideation or disproportionate psychological distress, a picture emerges of elderly residents being at relative ease compared to other study populations. They are not particularly worried or frightened of the future; with an endorsement of desire for death perhaps indicative of a readiness to die. This is consistent with most evidence that concludes fear of death tends to be greater among younger age groups and declines with increasing age [50–53].

While study group affiliation did not differentiate participants whose dignity was most likely to be compromised, various existential, psychological and to a seemingly lesser extent physical issues, distinguished those whose dignity was and was not intact. In other words, the intricacies of dignity seem best understood, not so much according to the specific condition moving someone towards death, but the constellation of issues imposed by said condition. Consistent with previous studies [15,17,54,55], losing dignity is affiliated with an assault on personhood, and feeling a diminished sense of worth or value; a burden to others; not feeling in control of one’s life; having unfinished business; worrying about the future, while expressing dissatisfaction with the past—along with constitutional symptoms, such as fatigue, constipation and weakness, which can further undermine autonomy and heighten physical dependency.

The primary limitation of this study is that it knowingly chose to examine four populations at a very specific point in time. Each group was identified according to criteria suggesting relative proximity to death and hence, the appropriateness of a palliative care approach. The study was designed to differentiate groups from one another, making them as mutually exclusive as possible. As such, all frail elderly participants were competent (a minority of residents in personal care homes) and older than 80 years of age; patients with ALS were adults up to 80 years of age; and patients with COPD or ESRD were 65 to 80 years of age, and thus more likely to have a more imminent terminal course. While these specific criteria may somewhat limit the generalizability of our findings, designing the study in this fashion was necessary to examine differences across these four non-cancer populations.

The PDI provides a novel way to examine the complexities of patient experience in these non-cancer populations. These populations can certainly be described as nearing death, given that 35–58% of them, depending on group affiliation, died during the course of the study. These deaths took place over a protracted period of months, hence supporting the rational for earlier integration of palliative care. Despite levels of distress comparable to patients with end-stage cancer, palliative approaches tend to be underutilized for non-malignant conditions or not offered at all. Applying the PDI across these four populations reveals unique constellations of symptoms and issues along distinct pathways leading to death. Knowing the intricacies of distress marking these pathways will broaden our understanding of end-of-life experience for these non-cancer populations and how best to meet their palliative care needs.
