# Correction: Dignity and Distress towards the End of Life across Four Non-Cancer Populations

**DOI:** 10.1371/journal.pone.0188141

**Published:** 2017-11-09

**Authors:** Harvey Max Chochinov, Wendy Johnston, Susan E. McClement, Thomas F. Hack, Brenden Dufault, Murray Enns, Genevieve Thompson, Mike Harlos, Ronald W. Damant, Clare D. Ramsey, Sara Davison, James Zacharias, Doris Milke, David Strang, Heather J. Campbell-Enns, Maia S. Kredentsere

There are errors in Table 2. The HADS scores included in the ALS, COPD, ESRD, FE, SISC DIGNITY < 3 (Intact), and SISC DIGNITY ≥ 3 (Fractured) columns are incorrect. Please see the corrected Table 2 here.

**Table 2:** Scores on Measures of Functioning Across the Four Study Groups; and Mean Levels of Functioning by Level of Dignity (Superscript indicates ranking of functioning between groups [1 = highest, to 4 = lowest].

	LEVEL OF DISTRESS ACROSS STUDY GROUPS [Mean (SD)]					SISC DIGNITY <3 (Intact)	SISC DIGNITY ≥ 3 (Fractured)	
	ALS	COPD	ESRD	FE	p-value^+^	Means (SD)	Means (SD)	p-value^+^
Mean Number of PDI Items Rated Problematic	6.2 (5.2)^4^	5.6 (5.9)^3^	2.3 (3.9)^1^	3.0 (4.4)^2^	< .0001	3.9 (4.9)	9.2 (5.6)	< .0001
ESAS-R								
Pain^a^	2.3 (2.6)^4^	3.5 (3.6)^1^	3.0 (3.2)^2^	2.7 (3.1)^3^	NS	2.8 (3.1)	4.1 (3.6)	-.07
Nausea^a^	0.5 (1.4)^4^	0.8 (1.8)^2^	1.0 (2.1)^1^	0.7 (1.6)^3^	NS	.07 (1.7)	1.0 (2.2)	NS
Drowsiness^a^	2.1 (2.7)^3^	2.4 (2.9)^2^	2.5 (2.8)^1^	1.6 (2.4)^4^	NS	2.1 (2.7)	3.2 (2.9)	.05
Shortness of Breath^a^	2.9 (3.0)^2^	6.0 (3.0)^1^	1.5 (2.3)^3^	1.1 (1.9)^4^	< .0001	2.8 (3.2)	3.5 (3.8)	NS
Anxiety^a^	2.4 (2.6)^2^	3.4 (3.4)^1^	1.7 (2.5)^3^	1.4 (2.3)^4^	< .0001	2.1 (2.7)	4.1 (3.6)	.003
Fatigue^a^	4.3 (2.9)^1^	4.2 (3.1)^2^	3.7 (3.1)^3^	2.6 (2.8)^4^	< .0001	3.6 (3.0)	6.0 (3.3)	.001
Constipation^a^	1.8 (2.8)^1^	1.6 (2.9)^2^	1.4 (2.6)^3^	1.8 (2.8)^1^	NS	1.5 (2.6)	3.8 (3.7)	.0002
Diarrhea^a^	0.5 (1.5)^3^	0.5 (1.8)^3^	0.9 (1.9)^1^	0.7 (2.0)^2^	.02	.65 (1.8)	.80 (2.3)	NS
Weakness^a^	5.6 (3.3)^1^	3.5 (3.1)^2^	3.2 (3.0)^3^	2.6 (2.9)^4^	< .0001	3.5 (3.2)	5.8 (3.4)	.001
Trouble sleeping^a^	1.9 (2.6)^3^	2.4 (3.4)^2^	3.2 (3.3)^1^	1.4 (2.5)^4^	.0001	2.2 (3.0)	3.1 (3.6)	NS
Dizziness^a^	0.6 (1.3)^3^	1.5 (2.5)^1^	0.9 (1.9)^2^	0.9 (2.0)^2^	.04	.9 (2.0)	1.2 (1.7)	NS
Difficulty thinking^a^	0.7 (1.5)^4^	0.9 (2.1)^3^	1.0 (2.0)^2^	1.3 (2.5)^1^	NS	.9 (2.0)	1.6 (2.4)	-.07
Will to live^b^	8.4 (2.9)^3^	9.2 (2.0)^1^	8.9 (2.3)^2^	8.4 (2.6)^3^	NS	8.7 (2.4)	8.0 (3.4)	NS
Appetite^b^	6.5 (3.4)^4^	7.5 (3.0)^2^	8.0 (2.5)^1^	7.3 (2.8)^3^	.009	7.4 (2.9)	5.7 (3.3)	.006
Activity^b^	4.0 (2.8)^4^	4.5 (2.7)^3^	6.0 (2.6)^1^	4.6 (2.9)^2^	< .0001	4.9 (2.8)	3.3 (3.5)	.01
Wellbeing^b^	7.3 (3.0)^4^	7.4 (2.7)^3^	7.8 (2.4)^2^	7.9 (2.6)^1^	NS	7.8 (2.5)	4.9 (3.2)	< .0001
No. ADL Dependencies^e^	2.2 (2.1)^2^	0.7 (1.2)^3^	0.4 (1.0)^4^	3.3 (1.5)^1^	< .0001	1.6 (1.8)	2.9 (2.2)	.002
Charlson comorbidity^f^	2.5 (1.5)^4^	5.3 (1.9)^3^	7.8 (2.0)^1^	7.1 (1.6)^2^	< .0001	4.6 (2.5)	5.8 (2.7)	.045
Hope Hearth Index (HHI)^c^	37.4 (4.7)^3^	37.8 (5.0)^2^	39.0 (5.5)^1^	35.9 (6.0)^4^	.008	37.8 (5.2)	34.0 (6.1)	.0008
Spirituality Belief Scale^d^	44.7 (19.5)^3^	43.9 (20.1)^4^	49.3 (22.2)^1^	48.4 (19.8)^2^	NS	47.2 (20.3)	37.8 (22.0)	.02
HADS^g^	5.6 (3.6)^1^	5.4(3.7)^2^	3.8 (3.1)^4^	4.6 (3.4)^3^	.001	4.7 (3.4)	7.5 (4.6)	< .001
MSPSS Social Support^h^	74.6^1^ (10.5)^1^	69.6 (12.0)^2^	69.4 (11.8)^3^	66.8 (12.4)^4^	< .0001	70.2 (11.9)	69.3 (14.0)	.01

^+^Kruskal-Wallis Comparison of Distributions

^a^ESAS-R item; scored 0 (no problem) to 10 (very significant problem)

^b^ESAS-R item; scored 0 (low/poor) to 10 (high/good)

^c^Hope Hearth Index; Scored 12 (least hope) to 48 (most hope)

^d^Spiritual Beliefs Scale; Scored 9 (unimportant/not worthwhile/rarely) to 90 (very important/very worthwhile/always)

^e^The Katz Index of Daily Basic Living; Scored 0 (independent in six functions) to 6 (dependent in six functions)

^f^Charlson Comorbidity Index; Number of Comorbid conditions

^g^HADS (depression items only); Scored 0 (normal/no depression) to 21 (severe depression)

^h^MSPSS (Multidimensional Scale of Perceived Social Support); Scored 12 (low social support) to 84 (high social support)
